# Introducing Motivational Interviewing in a Sickness Insurance Context: Translation and Implementation Challenges

**DOI:** 10.1007/s10926-017-9731-0

**Published:** 2017-08-30

**Authors:** Christian Ståhl, Maria Gustavsson

**Affiliations:** 1 Department of Medical and Health Sciences, Linköping University, 581 83 Linköping, Sweden; 2 Department of Behavioural Sciences and Learning, Linköping University, 581 83 Linköping, Sweden; 3 HELIX Competence Centre, Linköping University, Linköping, Sweden

**Keywords:** Motivational interviewing, Return to work, Rehabilitation, Social security, Insurance, Disability

## Abstract

*Purpose* Motivational interviewing (MI) is a conversational method to support clients in need of behavioral change. In an organizational reform, most Swedish sickness insurance officials were trained in MI to promote clients’ return to work (RTW) after sick leave. The aim of this article is to investigate experiences of introducing MI as a tool to promote clients’ RTW within a sickness insurance context, with special focus on the translation and implementation of the method. *Methods* A qualitative approach, comprising 69 interviews with officials, managers, and regional coordinators on two occasions. The material was analyzed through qualitative content analysis. *Results* Officials were positive about MI, but the application was limited to using certain tools with extensive individual variation. Officials struggled with translating MI into a sickness insurance context, where the implementation strategy largely failed to offer adequate support, due to low managerial priority, competing initiatives, and a high workload. Results of the educational intervention could therefore be seen on an individual but not an organizational level. *Conclusions* In order to translate MI into a sickness insurance context, training needs to be supported by organizational approaches that promote collective learning and sharing of experiences among officials. The results also illustrate how a method cannot be assumed to be implemented simply because training has been provided. Consequently, the application of the method needs to be carefully monitored in studies of interventions where MI is claimed to be used, in order to measure its effectiveness.

## Introduction

Motivational interviewing (MI) has been identified as a conversational method that may be useful for supporting patients or clients in need of behavioral change in the processes of rehabilitation and return to work (RTW) [1, 2], based on the notion that motivation is a central issue in rehabilitation [3, 4]. MI focuses on motivation and exploring ambivalence to change, and has been shown to be an effective method for promoting the willingness and ability to change behaviors [5], reducing barriers for change such as low self-esteem, and work out plans for personal development [6]. The method engages clients in conversations about behavior change, where fundamental components are four central processes: engaging, focusing, evoking, and planning [5, 7]. The processes aim to engage the client in order to evoke their inherent motivation to change a situation that has negative consequences for them, where the conversation focuses on what aspects of the situation that are possible to change, and on developing an action plan for how to achieve such a change, which should be tailored for each individual [5]. To do this, the person leading the conversation may make use of specific tools, e.g., self-evaluation rulers to assess readiness for change, and decisional balance worksheets where positive and negative aspects of changing the current situation are explored; MI further uses open-ended questions to evoke clients’ perspectives, and reflections, affirmations and summaries to provide feedback to the client throughout the conversation [7].

Commonly, MI is utilized by healthcare professionals to support patients or clients in need of behavioral changes such as smoking cessation or drug rehabilitation [5, 7]. Evidence of the method’s efficiency is relatively well established in certain areas, such as managing addictions, and insufficient in other areas, such as social work. It has been suggested that MI may be relevant in social work, although it presupposes a long relationship between the client and the professional [8]. However, Miller and Rollnik argue that MI is possible to use between clients and professionals with shorter relationships if the professional is sufficiently skilled in using the method and if it is utilized according to its fundamental components [7]. Other studies have tested MI in employment counselling, sometimes in combination with other methods such as Supported Employment [9, 10]. A recurrent conclusion is that the method—if applied correctly—is useful in employment counselling since ambivalence is often a problem for the unemployed. It is also noted that sufficient training in the method is crucial for success and that there must be enough time in meetings with the client to create trustful relationships. MI may also be used in situations where the client and the professional has a coercive relationship, as in correctional treatment [7]. In such cases, it is important that the goals and interests that MI is used to promote come from the individual, and not the enforcing institution.

Regarding the context of sickness insurance and RTW, recent studies have highlighted that MI may be efficient for RTW for musculoskeletal disorders, in combination with graded activity, therapeutic exercise and workplace accommodations [11, 12]. Apart from these results, there are few reliable studies of the effectiveness of MI in the processes of rehabilitation and RTW [1], apart from small experimental studies [13] and studies where MI was a part of a larger intervention [14–16]. Furthermore, studies of how to successfully train professionals in applying MI in the context of sickness insurance are lacking, as are studies on how MI is translated to and implemented in the context of sickness insurance on an organizational level.

As part of a larger organizational reform, the Swedish Social Insurance Agency (SSIA) decided to introduce MI as a method to be used by their insurance officials in meetings with people on sick leave. The aim of this article is to investigate experiences of introducing MI as a method to promote clients’ rehabilitation and RTW process within a sickness insurance context, with a particular focus on challenges concerning the translation and implementation of the method in the Swedish Social Insurance Agency.

### Research Setting

The Swedish sickness insurance system provides financial compensation through sickness benefits to people on sick leave. The Swedish Social Insurance Agency (SSIA) is the authority administering the system, and has around 13,500 employees in offices across the country, including 3500 officials who work with the administration of sickness insurance. Insurance officials have mixed educational backgrounds, but all who are recruited have a university education. Newly recruited officials also receive internal training in regulations and other tasks that are considered necessary to manage the professional role. The officials have a large responsibility for specific cases from the onset of sickness absence and in the process of rehabilitation and RTW. The insurance officials administer benefit payments and also coordinate the rehabilitation process, which involves contacts with stakeholders such as health care providers and employers.

The introduction of MI into the SSIA was part of a larger organizational reform, introduced to increase the client-orientation of the agency, and as a response to a trend of declining public trust in the agency. The reform consisted of the introduction of lean and team organizing, to facilitate a more customer-oriented service provision and to promote employee engagement in continuous improvements. Within this larger reform, MI was introduced as a tool for officials to use in their meetings with clients, in order to improve communication skills and client-orientation.

MI was introduced through an educational intervention that involved all insurance officials who had been working for at least 1 year in the agency. The education was specifically designed through an in-house competence center, where the purpose was to strengthen the competency of officials in having constructive meetings, and to support people to take control over their situation. Specific goals were that the official should understand the importance of their way of meeting clients, to be able to use the tools and strategies in MI, and to contribute to change through empathetic communication. A short book that introduced MI into the context of social insurance and the SSIA was handed out, which participants in the education were required to read. The officials were then given a 2 + 2-day course, provided by externally contracted educators who were certified MI instructors. The first 2 days of the course covered the basics of MI, i.e. the theoretical background and the various strategies and tools available. Between the course dates, the participants had homework, consisting of recording a meeting with a client, which was used for discussions at the next meeting. Hence, the education combined theoretical and practical elements with feedback from teachers and peers. After the course, the officials were instructed to continue recording client conversations (in meetings) where they applied the method, in order to get feedback from colleagues. To facilitate this, one official in each office was given the role as a coach for their colleagues to facilitate the application of MI, e.g., through listening to recorded conversations. The coaches were recruited internally among experienced officials, who were given additional training through a 3-day seminar and regional meetings. Regional coordinators were to support the coaches in this task. Unit managers were given a short introductory course to MI but had no specific task in supporting the implementation of MI. No specific measures were taken to monitor the use of or measure the effectiveness of MI.

Later, the SSIA decided to fund an evaluation of the MI intervention, which researchers were invited to apply for. The evaluation, which this article reports from, was hence initiated after the educational intervention had been carried out.

## Methods

The study used a qualitative approach in which interviews were carried out with insurance officials, managers, and regional coordinators in the SSIA. Two sets of interviews were carried out in different stages of the implementation process—the first soon after the educational intervention, and the second a year later (Table 1).

**Table 1:** Overview of data material in the study

Time for data collection	Participants in the interviews
May–September 2013	Unit managers, n = 20 Regional coordinators, n = 4 Officials, n = 24
May–September 2014	Unit managers, n = 5 Officials, n = 16

The selection of respondents was made to achieve geographical representation, where interviews were carried out in four offices in medium-sized cities in the north, south, east and west parts of Sweden. Unit managers were approached with an invitation to the study, and were asked to invite officials to participate and to organize a schedule for the interviews, which were carried out during a few days in each office. Since the sample was based on offices, the ambition was to interview a number of officials (including the official selected to coach his/her peers) and the unit manager, to get a comprehensive description of how MI was used in the specific office. This resulted in a relatively large number of interviews, where the first set of interviews included 24 officials and six unit managers. In addition, four regional coordinators and 14 more unit managers from other offices were interviewed, to achieve a description of how MI was implemented in other regions. The interviews with officials focused on their experiences of MI as a method of talking to clients in their daily work, how the educational training was perceived, how support in applying the method had worked, and whether or not they considered the method to be applicable in a social insurance setting. The interviews with managers and coordinators focused on the implementation of MI, how it was introduced into working routines, and which strategies were used to sustain and develop the use of the method in daily work. Interviews were carried out in the participants’ offices, with the exception of three managers and one coordinator who were interviewed over the phone.

The second set of interviews was carried out 1 year after the first, at the same four offices with the same officials and managers. The focus in the follow-up interviews was to ascertain whether the officials were still using MI, and how they perceived their competence in using the method. Managers were asked about strategies and measures taken to maintain relevant competence in MI within the organization.

Interviews were carried out by a research assistant, in continuous dialogue with the principal investigator (CS) to maintain rigor throughout the process. Interviews were recorded and lasted for between 45 and 60 min, and were transcribed verbatim.

### Data Analysis

The interviews were analyzed through a qualitative content analysis [17], carried out by the two authors. In the first step, each interview was read separately, and categorized through the identification of meaning units. Through the categorization process, two overarching categories emerged: (1) implementation of the method in a sickness insurance agency; and (2) using MI in a sickness insurance context. Both individual and organizational aspects were scrutinized. In the second step, differences and similarities were analyzed among officials and managers as separate groups, with reference to the categories identified. After this inductive analysis, the results were related to institutional theories on translation of ideas, and organizational development theories. These theories were identified with regard to the characteristics of the results, where issues of implementing MI into a new context was identified as central, as was organizational challenges in making this work in practice.

Due to the large amount of data and the space constraints of the article, results are presented in a relatively summarized way, focusing on a descriptive report of the issues related to implementing and using MI. A more detailed presentation of the results is given in two Swedish reports [18, 19], and an additional forthcoming article which will focus on how the organizational reforms have influenced the role of the insurance official.

### Ethical Considerations

All participants were informed about the purpose of the study and that they could withdraw their participation at any time. The project was approved by the regional ethics board in Linköping (dnr 2013/83-31).

## Results

The results are organized into two broad categories: (1) implementing MI in a sickness insurance agency; and (2) using MI in a sickness insurance context.

### Implementing MI in a Sickness Insurance Agency

Several organizational conditions were identified as obstructing the implementation of MI into the organization: the strategy for introducing the method, support from the management, feedback on the use of MI, workload, and priority within the SSIA.

The strategy for introducing MI was based on an educational intervention and a time-limited workplace-based support system in which certain officials were to coach their colleagues in applying the method. The strategy, however, failed to include line managers as central actors in the implementation. As a result, a vast majority of officials perceived managers as being unsupportive, and using MI was not seen as an organizational priority. The interviewed officials stated how their managers had been highly unsupportive in relation to MI, and that they appeared disinterested in whether or how officials used MI in their daily work. The general perception was that it was up to the individual official whether they wanted to use the method or not.

They [managers] could have been more active in following this up. What they did was basically to buy this intervention and then not bother at all about how we worked with it. (Insurance official 4, office 2)

The managers themselves were self-critical and admitted that they had not focused on MI. This, some managers pointed out, was partly because they did not know enough about the method, which complicated supporting their employees in using it.

We managers had a 1-day summary of what our officials had learned, once they were finished with the course. It might have been better with the opposite so that I could have given more feedback and asked questions. (Unit manager 2)

Another organizational condition for using MI was workload, as it affected the practical possibilities of finding the time for training. Managers and officials alike emphasized that their current workloads were problematic. Officials pointed out that their caseloads made it unrealistic to expect anything more than fundamental administration (i.e., paying out benefits), which made the application of an additional method not yet embedded in routines difficult. Using MI was also perceived as requiring a series of meetings, which is normally not offered to clients.

We have too much to do. To work this way with follow-up and good meetings, with clients and other stakeholders; it takes a lot of time. A lot of time is needed for preparation – the meeting itself and follow-up meetings – at the same time as we have all of these other things to manage. So, our conditions don’t allow us to do more of anything. (Insurance official 4, office 4)

The lack of priority given to MI within the organization was another issue. During the same period as MI was being implemented, the SSIA was introducing lean and team organizing, which increased the strain on daily practice. Managers tended to consider these three initiatives (lean, teams, and MI) as part of a larger development strategy:

If you think about the overall vision of the SSIA, I see a clear connection. [...] It’s all about client-orientation in general, both practically and culturally. (Unit manager 13)

Officials, however, perceived no such connection; rather, each initiative was seen as separate and requiring attention at the cost of the others, and the implementation of lean and teams was perceived by officials as overshadowing the focus on MI. Lean and teams were reforms of how the daily work was organized and hence not possible for officials to keep from. Practicing MI, on the other hand, was more up to the individual. When pressured, officials would prioritize administering benefits, rather than spending time learning new methodologies.

Both managers and officials found it difficult to say how common the use of MI was, both in the local offices and in the SSIA as a whole. The managers had no estimation of the extent MI was being used, which indicates that there were no routines for following up whether the method was used or not. While officials testified about effects in their daily work, they did not know whether or not their colleagues were using MI.

There is no way to know what the others do as we seldom have meetings together. [...] So we don’t really know about each other, I just know what I do. (Insurance official 2, office 2)

The use of MI could only be seen in the local context and was limited to individual meetings where officials had made changes to their work based on their own interpretation of the method. As a result, it is questionable whether it can be said that MI has been implemented into the organization.

### Using MI in a Sickness Insurance Context

Most of the officials appreciated MI as a relevant idea and a tool for conducting meetings with people on sick leave. The officials described that the use of MI constituted an open approach that prompts clients to increase their insight into their situation, and hence taking more individual responsibility for their rehabilitation process. Officials also described how MI had helped them to listen to clients, making meetings more effective.

Instead of leaving a meeting and taking all the problems with me, I have started processes within the client, which is effective. (Insurance official 4, office 2)

Officials considered MI to be helpful when having to deliver negative decisions, such as denied benefits, as using MI principles made these meetings more dialogue-oriented and positive. It also served to open up for a discussion about different ways forward after such a decision.

Other officials were critical of the applicability of MI in a sickness insurance context, where one aspect was that the use of MI tools could make the client share more information than the official is able (or supposed) to manage. Hence, using MI may lead to expectations which officials are not able to fulfil. The sickness insurance legislation sets the framework for what options are available to the client in the rehabilitation process, and using MI may lead to discussing choices that are not allowed by the system. It is possible, from the insurance system’s perspective, to make the wrong choices in these cases.

The utilization of MI was described as irregular and was often limited to certain techniques. Several officials noted that they had lost the MI vocabulary, and therefore could not label the techniques they were using, apart from the more basic tools such as using open questions, reflections, and summaries. Other tools were rarely mentioned. Overall, many officials seemed to have adopted a certain “MI spirit”, without consciously applying specific techniques. MI appeared to be used primarily in physical meetings, which limits its applicability since the majority of officials’ contact with clients takes place over the phone or through e-mail.

To apply MI fully, you need to meet clients more often than we typically do. We usually don’t meet them that many times. (Regional coordinator)

Some officials felt insecure, uncomfortable, and unaccustomed in utilizing MI in their work. Despite having opportunities for using MI in their meetings with clients, doing so would require training and feedback, of which there was a lack. Some officials were concerned about whether they were applying MI correctly, due to their insufficient experience and competence in the method.

I use... well, parts of it, rather often. But usually smaller things that I picked up in the MI course, which isn’t really MI. I may choose to ask more open questions or to repeat what the client says, for example. (Insurance official 3, office 1)

The results also illustrate how translating MI into a sickness insurance context may be challenging, as some officials struggled with applying behavior change rhetoric to situations where the problem was perceived as being something else (e.g., disease or ill health). This represents translation issues on a conceptual level, where the focus and content of the method were perceived (by some) as being difficult to combine with organizational objectives.

There were also organizational aspects that obstructed the translation of MI into practice. Officials had little support in applying MI in their daily work, and the responsibility for translating MI into sickness insurance practice was, therefore, dependent on officials’ individual interpretations of how MI fits into the current practices. The interpretation of MI as promoted by the organization (e.g., through a book contextualizing MI into sickness insurance) was not enacted through managerial support or training.

## Discussion

The results identify several challenges related to applying MI in a sickness insurance context. We will in this section proceed to analyze these results using institutional theories on the translation of ideas, and theories on developmental work in organizations.

### Translation Challenges

Organizations are constantly changing through processes of imitation and translation [20]. Imitation is a key notion in how organizations approach and choose among ideas; some ideas appear to be fashionable and attractive because other organizations are applying them, and no organization wants to be unfashionable or backwards [21]. Hence, ideas are adopted on a basis of appropriateness, based on which ideas are currently being promoted in a certain organizational field [20]. Ideas are then translated into the new context through processes of dis-embedding (i.e. de-contextualizing the concept or idea from its original source) and re-embedding (re-contextualizing it into the current organizational context) [20, 22, 23], in which ideas shift into enacted practices. Such translations are formed by the institutional setting of the organization in which the idea is re-embedded, serving as rules for how the idea may be transformed and edited [20]. In the re-embedding phase, the idea may change with respect to its focus, content, and meaning [20] and can, therefore, be quite different to how it appeared in the original setting.

MI originates in healthcare and is still fairly new in the context of sickness insurance and promoting RTW. The organization’s choice to adopt MI as part of their strategy to improve public trust may be seen as an attempt to adhere to a broader trend of promoting client- or customer-oriented approaches to service delivery, imitating other organizations in a process of isomorphism [20, 21]. The translation of MI was initiated by the SSIA on an organizational level, re-embedding it as a conversational tool aiming to place more responsibility for rehabilitation on the individual on sick leave. The results show that some officials embraced this as a relevant and useful technique, while others struggled with applying the MI rhetoric to complex sick leave cases where motivation was not considered to be the main issue.

The focus on MI was part of a larger development process, where officials were to focus less on bureaucratic work routines and become more client-centered, i.e. moving from being an administrative official to being a case worker who builds relationships with clients. Since sickness benefits are one of the largest social insurance schemes in Sweden, it is perhaps unlikely that officials will be able to change their way of working to the extent that MI becomes relevant as a tool: many of the work tasks are administrative and have little to do with motivating clients. The MI approach also makes it possible for clients to make decisions about their rehabilitation that are not in line with what the insurance system requires or proposes, which may complicate communication between clients and officials if the latter are expected to promote specific organizational goals. Hence, the translation of MI into organizational practice was obstructed by problems related to the re-embedding of the MI idea into a bureaucratic organization.

### Implementation Challenges

Development strategies may be designed top-down, bottom-up, or through a horizontal approach based on cooperation and networks. Top-down strategies are based on a linear approach where control and measuring outcomes are used to monitor the change. Such strategies may lead to sustainable change if carefully implemented, although they tend to be inflexible. Bottom-up strategies are based on incremental changes by changing practice in small steps leading to quick results that are easy to implement [24]. Such changes are often limited in scope, but they may initiate larger development spirals. Literature recommends an integration of approaches to counter the limitations of top-down and bottom-up strategies. In order to successfully change organizational practices, changes need to be anchored in the organization in order to secure support and legitimacy on a managerial level, as well as acceptance and participation from first-line managers and employees [25]. Generally, changes that are targeting individual behavior will have effects that are limited to individuals, while changes that take organizational functioning into account have better prospects for influencing how the organization operates [25].

Another issue is the need to balance the processes of production and development [26]. Time for learning is often limited, and developmental work is often separated from daily operations, which limits the use of the workplace as a learning environment [26]. Line managers need to be engaged in learning and development activities that are supported by the top management [26, 27]. Lack of managerial support is a recurrent reason for failure in sustaining developmental work [25]. Managers have an important role in creating spaces and making time for reflection and feedback in their daily work, which is necessary to support competence development and the learning of new methods. Lack of this support will worsen conditions for development [28], and there is a plethora of studies from various sectors that identify lacking engagement or support from managers as a cause for failure on organizational change initiatives [29–31]. Managers influence whether or not the workplace becomes a development-oriented learning environment [32]. Employees may also support each other by initiating and requesting developmental activities [33], which requires employees to be motivated and have the skills, competencies, and self-confidence needed for trying out new ways of working [34]. A learning environment offers possibilities to engage in formal and informal learning activities, and the experiences from daily work may be used to develop how the organization operates. Formal learning activities, e.g., educational efforts, also need to be linked to daily practice in order to sustain changes within the organization.

The implementation of MI into the SSIA was irregular at best, which was largely due to a flawed implementation strategy. The strategy was designed using a top-down approach, and carried out through a large-scale educational intervention targeting all officials. The implementation strategy had bottom-up elements, where the application of MI was to be supported through peer-support in local offices, although this was designed top-down and not given the proper time and managerial support to work well. Peer support was not prioritized or followed up by managers, leaving the responsibility for translating and applying the method on individual officials. Officials had limited translational competence with regard to MI, and the implementation strategy had too little focus on workplace learning to deal with the issues of translation and adaptation. Officials expressed much criticism regarding how the SSIA managed the relatively large investment in MI, especially that the educational intervention had not been followed up in any organized way, and that training was not provided to develop and sustain competence in using the method. Officials were also critical to their possibilities to use the method related to their current work situation, where heavy caseloads implied little or no time for reflection and learning. As a result, it was not possible to identify any results from the implementation of MI on the organizational level; there were no indications of changes in routines for meetings with clients, and continuous monitoring of the use of the method was lacking. Furthermore, managers did not appear to be involved in supporting continuous use of and development of MI within the organization.

Interventions aimed at individuals generally lead to individual results, and more seldom to organizational development. In order to promote an organizational learning process, it is important for educational interventions to be clearly linked to the learning environment in daily practice [26–28]. To promote such a development, first-line managers need to be considered as strategic resources that should be used to support both the officials and the management in a more integrated competence development strategy [28].

## Conclusions

The application of MI in a social insurance context was challenged by translation and implementation difficulties, where insurance officials needed support in how to use the method to promote RTW for their clients. The organization largely failed to provide such support, leaving the translation task to the officials, who lacked the relevant competencies and conditions. Consequently, while MI training resulted in some officials changing the way they met with clients, changes on an organizational level were harder to identify.

These results are relevant in a RTW setting since they illustrate the importance of adequate training and support when introducing new methods, where even ambitious educational interventions may fail due to organizational negligence in sustaining its effects. An implication of these results is that it is not given that a method will be implemented just because training has been provided. MI has been described as a method that is simple but not easy [35], which means that while it may be easy to learn the basic idea, it is difficult to master the technique. Consequently, the practical work needs to be carefully monitored in studies of interventions where MI or other methods are claimed to be used, in order to measure their effectiveness.
